# HCCR-1, a novel oncogene, encodes a mitochondrial outer membrane protein and suppresses the UVC-induced apoptosis

**DOI:** 10.1186/1471-2121-8-50

**Published:** 2007-11-28

**Authors:** Goang-Won Cho, Seung Min Shin, Hyun Kee Kim, Seon-Ah Ha, Sanghee Kim, Joo-Hee Yoon, Soo Young Hur, Tae Eung Kim, Jin Woo Kim

**Affiliations:** 1 Molecular Genetic Laboratory, College of Medicine, The Catholic University of Korea, Seoul 137-040, Korea; 2 Department of Obstetrics and Gynecology, College of Medicine, The Catholic University of Korea, Seoul 137-040, Korea

## Abstract

**Background:**

The Human cervical cancer oncogene (HCCR-1) has been isolated as a human oncoprotein, and has shown strong tumorigenic features. Its potential role in tumorigenesis may result from a negative regulation of the p53 tumor suppressor gene.

**Results:**

To investigate the biological function of HCCR-1 in the cell, we predicted biological features using bioinformatic tools, and have identified a LETM1 homologous domain at position 75 to 346 of HCCR-1. This domain contains proteins identified from diverse species predicted to be mitochondrial proteins. Fluorescence microscopy and fractionation experiments showed that HCCR-1 is located in mitochondria in the COS-7, MCF-7 and HEK/293 cell lines, and subcompartamentally at the outer membrane in the HEK/293 cell line. The topological structure was revealed as the NH_2_-terminus of HCCR-1 oriented toward the cytoplasm. We also observed that the D1-2 region, at position 1 to 110 of HCCR-1, was required and sufficient for posttranslational mitochondrial import. The function of HCCR-1 on mitochondrial membrane is to retard the intrinsic apoptosis induced by UVC and staurosporine, respectively.

**Conclusion:**

Our experiments show the biological features of HCCR-1 in the cell, and suggest that uncontrolled expression of HCCR-1 may cause mitochondrial dysfunction that can result in resisting the UVC or staurosporine-induced apoptosis and progressing in the tumor formation.

## Background

Mitochondria are responsible for a number of metabolic tasks in eukaryotic cells. Their primary function is to generate energy through oxidative phosphorylation in organelles. Mitochondria also play an important role in other biological activities such as programmed cell death [1], calcium signaling and generation of detoxification of reactive oxygen species [2].

Mitochondria consist of four subcompartments – an outer membrane, an inner membrane, an inter-membrane space and matrix. The chaperone proteins, and the translocase components, help mitochondrial transport into the subcompartments through their membranes [3]. During the cytoplasmic translation of mitochondrial proteins, they are recognized by chaperone proteins which interfere with the folding of the protein. A positively charged signal sequence, on the mitochondrial protein, then binds to the translocase components called translocase of the outer mitochondrial membrane (TOM) located on the outer mitochondrial membrane [4,5], the protein is moved into the TOM complex. Translocase of the inner mitochondrial membrane (TIM) [6,7], is an inner membrane protein complex that forms a pore, and acts to pull the mitochondrial protein through the pore. On the inside of the pore, different chaperone proteins recognize the mitochondrial protein and fold it into a functional form. The signal sequence is removed by a mitochondrial signal peptidase as the mitochondrial protein enters the matrix.

The leucine zipper-, EF-hand-containing transmembrane protein 1 (LETM1) is one of the mitochondrial proteins that is posttranslationally imported into the mitochondrial inner membrane [8]. This gene has been identified from the Wolf-Hirschhorn syndrome (WHS) [9] which is characterized by multiple congenital anomalies, severe pre- and post-natal growth retardation and mental retardation [10,11]. In almost all patients with WHS, LETM1 is part of a deletion found at the chromosome 4 location [9]. Its role in WHS may be based on mitochondrial K^+ ^homeostasis [12].

The human cervical cancer oncogene (HCCR) has been isolated and identified as a human oncoprotein [13]; it has revealed strong tumorigenic features in experiments on nude mice [13]. Its role in tumorigenesis may be negative regulation of the p53 tumor suppressor gene [13]. HCCR-transgenic mice have been shown to develop breast cancers and metastasis [14]. In order to investigate the functional role of HCCR-1, we predicted the biological features using bioinformatic tools. In addition, we identified the subcellular location and the targeting signal of HCCR-1.

## Results and Discussion

### HCCR-1 has a LETM1 homologous domain, and is predicted to be a mitochondrial protein

**Figure 1:**
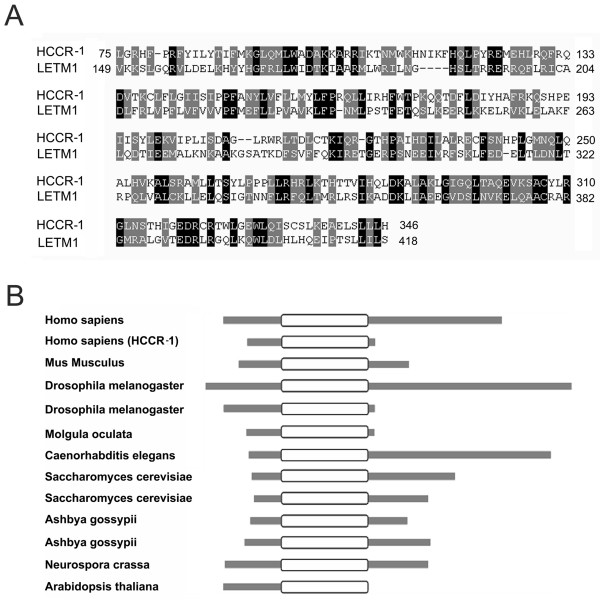
**Alignments of HCCR-1 with its homologs**. (A) Alignment of HCCR-1 with Human LETM1 protein (gi18204589 ref AAH21208). Identities are highlighted in black and conserved substitutions by gray. (B) Schematic alignment shows the LETM1 domain structure. Analysis using Pfam revealed sequence similarity with the LETM1 domains. Scale is lengths proportional to their molecular masses. The locations of putative LETM domains are indicated by blank boxes. Homo sapiens gi18204589 ref AAH21208; Homo sapiens (HCCR-1) gi13624098 ref AAK34885; Mus musculus gi33416955 ref AAH55685; Drosophila melanogaster gi21626751 ref AAM68317; Drosophila melanogaster gi1749774 ref CAA71125; Molgula oculata, gi308969 ref AAC37181; Caenorhabditis elegans, gi17561658 ref NP_506382; Saccharomyces cerevisiae, gi6324546 ref NP_014615; Saccharomyces cerevisiae, gi1762146 ref AAB70096; Ashbya gossypii gi44980464 ref AAS50397; Ashbya gossypii, gi44980448 ref AAS50381; Neurospora crassa gi32420419 ref XP_330653; Arabidopsis thaliana, gi42562974 ref NP_176732.

Since HCCR-1 has been shown to have tumorigenic features previously [13,14] we focused on the biological functions of HCCR-1 in the cell. In order to determine the functional domain of HCCR-1 at the molecular level, Pfam version 18.0 was used to analyze its amino acid sequences and identified with the LETM1 homologous domain at amino acids 75 to 346 of HCCR-1 (Fig 1A). Sequence alignment between the domain from HCCR-1 and from the LETM1 protein using CLUSTALW [15] showed a 57.61% sequence similarity. From the analysis using Pfam, we also identified several LETM1 domain-containing proteins, in various eukaryotic species, from human through fungi as well as plant (Fig 1B); these findings suggest that the domain has widespread application in organisms and is well conserved during evolution, and that the LETM1 domain may have an important biological function in the normal life cycle.

Since some of the LETM1 domain-containing proteins have been identified as mitochondrial proteins [8], we analyzed the subcellular localization of the LETM1 domain-containing proteins using the TargetP version 1.1 [16] and Predotar version 1.03 [17]. As shown at Table 1, almost all proteins were predicted to be mitochondrial proteins with a high score (Table 1). It strongly suggests that LETM1 domain may play an important role in mitochondria.

**Table 1:** Prediction of mitochondrial targeting of HCCR-1 and LETM1 domain-containing proteins.

		TargetP		Predotar
			
		Score	RC	Score
Human	AAH21208	0.882	2	0.74
	AAK34885	0.898	1	0.39
Mouse	AAH55685	0.442	4	0.04
Fly	AAM68317	0.932	2	0.04
	CAA71125	0.861	2	0.72
Squirt	AAC37181	0.670	4	0.68
Worm	NP_506382	0.943	2	0.84
Fungi	NP_014615	0.924	1	0.75
	AAB70096	0.822	3	0.78
	AAS50397	0.934	1	0.85
	AAS50381	0.920	1	0.81
	XP_330653	0.798	2	0.10
Plant	NP_176732	0.634	4	0.09

Mitochondrial targeting sequences were detected by TargetP version 1.1 [16] and Predotar version 1.03 [17]. TargetP calculates relative for mitochondrial localization. The reliability classes (RC) of TargetP provide a score from 1 to 5, where 1 indicates the strongest prediction. Predotar provides a probability estimate as to whether the sequence contains a mitochondrial targeting sequence. Proteins with scores above 0.5 are very probably targeted to the mitochondria.

### HCCR-1 is expressed on the mitochondrial outer membrane

**Figure 2:**
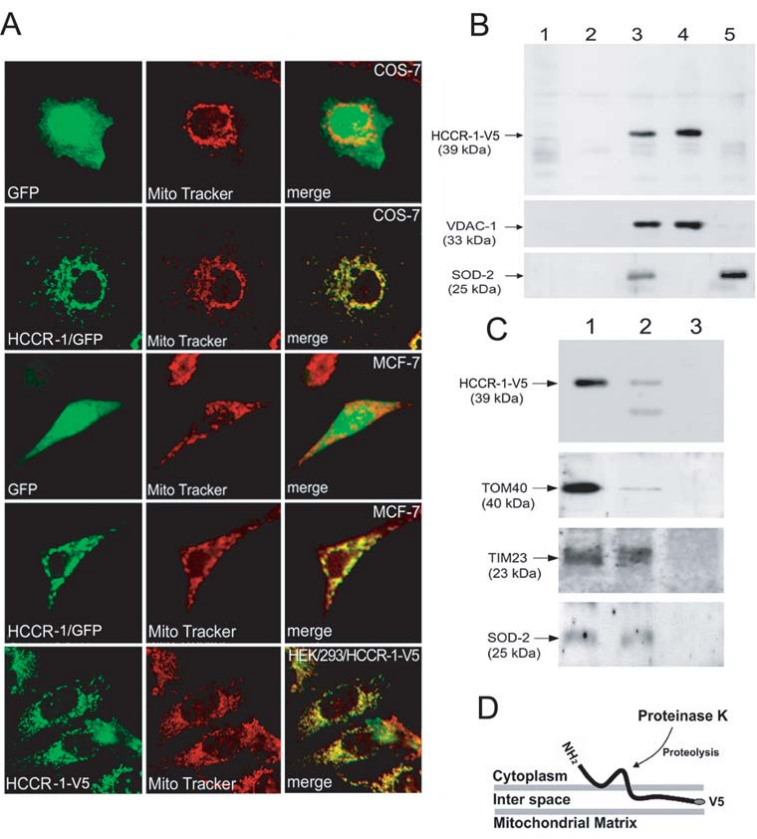
**HCCR-1 is localized on the mitochondria outer membrane**. (A) COS-7 (top and second panels), MCF-7 (third and forth panels) and HEK293/HCCR-1-V5 (bottom panel) cells were cultured on the pre-coated coverslip for overnight. COS-7 and MCF-7 cells were transiently transfected with the pEGFP-N1 vector (GFP) or the vector carrying the human HCCR-1 gene tagged with GFP at its COOH-terminus (HCCR-1/GFP). The cells were incubated with MitoTracker (Red), fixed with 4% paraformaldyhyde. HEK293/HCCR-1-V5 cells were stained with MitoTracker (Red) and fixed with 100% methanol for 1 min at -20°C. Mouse anti-V5 antibody (1:300 dilution) and goat anti-mouse IgG conjugated with HRP (5 μg/ml) were applied to the cells for 1 h at room temperature (HCCR-1-V5). The cells were mounted and examined using a confocal microscope. (B) HCCR-1 is expressed at the mitochondrial membrane in the HEK293 cell line. Subcellular fraction of HEK293/HCCR-1-V5 cell line. HEK293/HCCR-1-V5 cells were fractionated into the nuclei (lane 1), post-mitochondria (lane 2), and mitochondria (lane3) fractions by differential centrifugation. Isolated mitochondria were treated with 0.1 M Na_2_CO_3 _and fractionated by centrifugation at 100,000 g into pellets (lane 4) and supernatant (lane 5). Proteins were analyzed by immunoblotting using antibodies to the V5 epitope (top panel), the mitochondrial membrane protein VDAC-1 (middle panel) and the mitochondrial matrix protein SOD-2 (bottom panel). (C) HCCR-1 topology on the mitochondrial outer membrane. Intact mitochondria were incubated for 30 min at room temperature without (lane 1) and with 50 μg/ml proteinase K (lane 2) or with 50 μg/ml proteinase K plus 1% Triton X-100 (lane 3). Each sample was precipitated with TCA and separated by SDS-PAGE and analyzed by immunoblotting with antibodies to V5 (top panel), to the outer membrane protein TOM40 (second panel) and to the inner membrane protein TIM23 (third panel), and to the matrix protein SOD-2 (bottom panel). (D) Schematic diagram shows the HCCR-1 topology at the outer mitochondrial membrane.

In order to examine the subcellular localization of HCCR-1, cDNA was subcloned into a pEGFP-N1 vector. COS-7 and MCF-7 cells were cultured on a pre-coated coverslip and transiently transfected with the pEGFP-N1 vector (Fig 2A, GFP) or the vector carrying the human HCCR-1 cDNA (Fig 2A, HCCR-1/GFP). The cells were then further incubated for 24–28 h and their mitochondria were stained with MitoTracker for 15 min at 37°C. The cells were fixed, mounted, and analyzed by confocal microscopy (Fig 2A, top through forth panels). We also used the HEK293 cell line which stably expresses the V5 tagged HCCR-1 (Fig 2A, HEK293/HCCR-1-V5). The cultured cells on the pre-coated coverslip were stained with MitoTracker, fixed with 100% methanol for 1 min at -20°C. Mouse anti-V5 antibody and goat anti-mouse IgG antibody conjugated with horseradish peroxidase (HRP) were applied to the cells for 1 h at room temperature. The stained cells were mounted and analyzed by confocal microscopy (Fig 2A, Bottom panel). The HCCR-1 fluorescence images revealed a staining pattern resembling mitochondria in all tested cell lines (Fig 2A, HCCR-1/GFP and HCCR-1-V5). The visualized mitochondria, as a result of incubation with a mitochondria-specific dye (Fig 2A, MitoTracker), were overlaid with HCCR-1/GFP or HCCR-1-V5 (Fig 2A, merge) while the vector alone (Fig 2A, GFP) did not merge with the mitochondria; these findings suggest that HCCR-1 is localized at the mitochondria.

Sequence analysis of the HCCR-1 protein, using the Hopp-Woods method of calculating hydrophilicity and TMHMM Server Version 2.0 [18], revealed the presence of two hydrophobic segments, one extending from 139 to 161 residues which was predicted a transmembrane domain (TM) and the other extending from position 81 to 98 amino acids which may be involved in anchoring to a membranous component. Therefore, HCCR-1 is predicted to be an integral membrane protein.

To examine whether HCCR-1 is a mitochondrial membrane protein, the HEK293/HCCR-1-V5 cell lines were studied for subcelllular fractionation experiments. Nuclear (Fig 2B, lane1), post-mitochondrial supernatant (Fig 2B, lane2), and mitochondrial (Fig 2B, lane3) fractions were separated by differential centrifugation. The isolated mitochondria were solubilized by treatment with alkaline sodium carbonate [19] and ultra-centrifuged to separate the soluble proteins (Fig 2B, lane 5) from the insoluble integral membrane proteins (Fig 2B, lane 4). Each of the fractionated proteins were displayed by SDS-PAGE and analyzed by immunoblotting using antibodies to the V5 epitope (Fig 2B, top panel), voltage dependant anion channel 1 (VDAC-1) which is expressed on the mitochondrial membrane [20] (Fig 2B, middle panel) and superoxide dismutase-2 (SOD-2) on the mitochondrial matrix [21] (Fig 2B, bottom panel). The HCCR-1 protein was found in the mitochondrial fraction (Fig 2B, lane3) which is consistent with the previous result shown in figure 2A. Treatment with alkaline sodium carbonate did not solubilize the mitochondrial integral membrane protein VDAC-1 (Fig 2B, middle panel, lane 4), but did disrupt the mitochondrial membrane as a result of exposing the matrix protein SOD-2 (Fig 2B, bottom panel, lane 5). HCCR-1 protein also remained in the pellet fraction (Fig 2B, top panel, lane 4); these findings suggest that HCCR-1 is exclusively localized in the mitochondrial membrane.

Since HCCR-1 was localized to the mitochondrial membrane, we evaluated the topological structure of HCCR-1. Bioinformatic analyses using the TMHMM Server Version 2.0 [18], showed that the NH_2_-terminus of HCCR-1 is exposed to the cytoplasm, and also predicted that HCCR-1 is a type II integral membrane protein.

To examine the HCCR-1 topology, the intact mitochondria were isolated from HEK293/HCCR-V5 cell lines and incubated with and without proteinase K or proteinase K plus Triton X-100. Each treatment was subjected to SDS-PAGE and visualized by immunoblotting using antibodies to the V5 epitope (Fig 2C, top panel), TOM40 which expresses on the outer mitochondrial membrane [4,5] (Fig 2C, second panel), TIM23 on the inner mitochondrial membrane [6,7] (Fig 2C, third panel), and SOD-2 on the mitochondrial matrix (Fig 2C, bottom panel). TIM23 and SOD-2 proteins were protease resistant in the mitochondria, which indicate that these proteins were not exposed to the surface. By contrast, Tom40, an outer membrane protein protruding to the surface, was sensitive to proteinase K and weakly detected by its polyclonal antibody (Fig 2C, lane 2). Interestingly, the immunoblot analysis, using anti-V5 monoclonal antibody, detected two sized proteins – a full sized HCCR-1-V5 (about 39 kDa) and a little smaller sized protein (about 32 kDa); this indicates that the NH_2_-terminus of HCCR-1 was partially degraded by the proteinase K, while the COOH-terminus of HCCR-1, containing V5 epitope, was protected from the protease activity in the intact mitochondria. This suggests that HCCR-1 is located on the outer mitochondrial membrane (Fig 2C). This result also suggests that the topological structure of HCCR-1 is indeed consistent with the bioinformatic prediction as shown in figure 2D. Upon disintegration of the membrane proteins by treatment with Triton X-100, all proteins were digested by proteinase K, showing that none of them was intrinsically protease-resistant (Fig 2C, lane 3).

### Mitochondrial targeting sequences are in the NH_2_-terminus of HCCR-1

The targeting signal, on mitochondrial proteins, often exists on their DNA fragments coding for the amino terminal region of the protein [22-24] and does not have a conserved sequence, but rather share a common physical property. This feature is the ability to form an amphipathic α-helix, one in which positively charged amino acids are localized to one surface of the helix [25-27]. This signal sequence is recognized by the receptors on the mitochondrial outer membrane and imported into mitochondria. We have shown that HCCR-1 is also a mitochondrial protein. This protein may be posttranslationally transported to mitochondria and then imported into mitochondria by recognition of the signal sequence.

**Figure 3:**
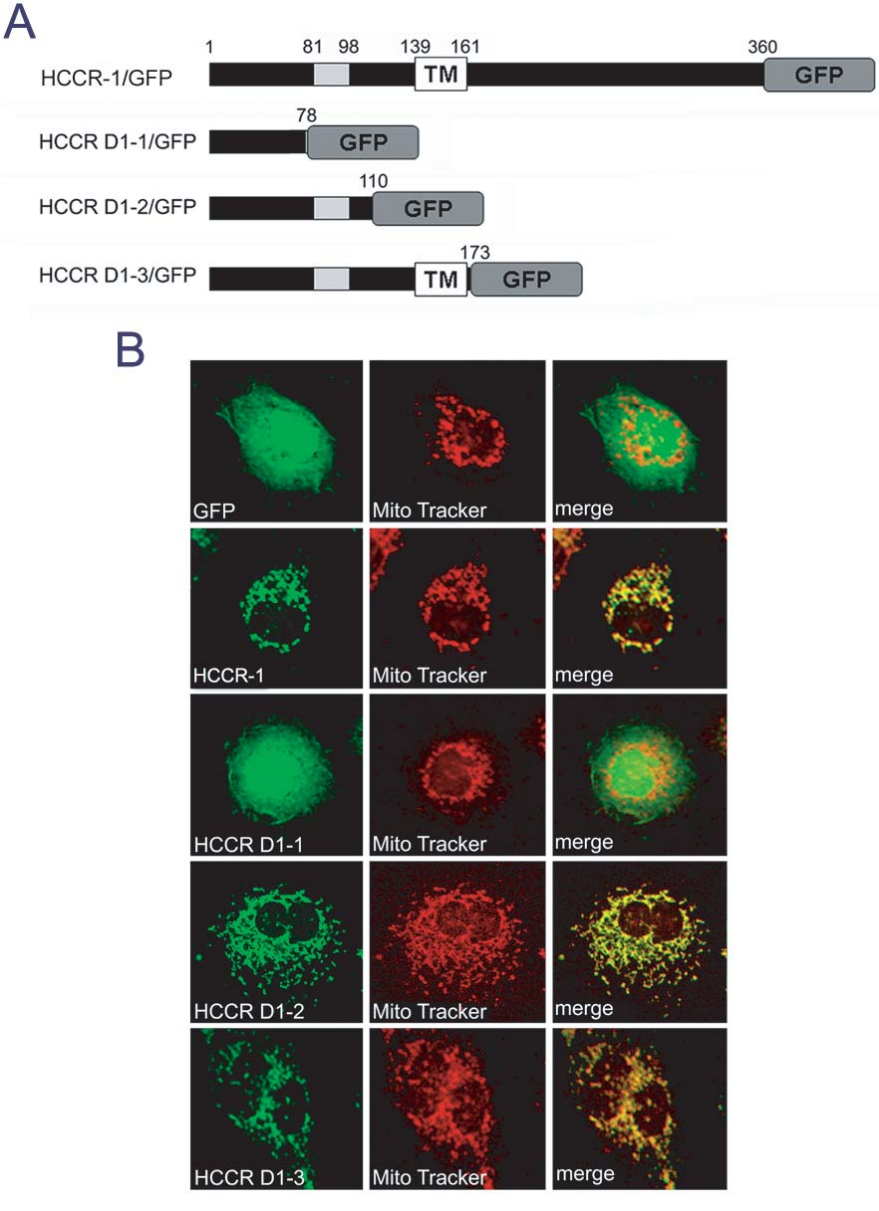
**NH_2_-terminus of HCCR-1 contains the mitochondrial targeting information**. (A) Schematic structure of HCCR-1 and its deleted mutants which were COOH-terminally linked by the GFP reporter gene. Grey boxes indicate the hydrophobic region. TM boxes indicate the locations of putative transmembrane domain. (B) Expression of wild-type or deleted mutants in COS-7 cells. COS-7 cells were transfected with the indicated constructs in the expression vectors. The cells were incubated with MitoTracker and fixed. Fluorescent images of GFP (green), MitoTracker (red) were taken using a confocal microscope. Merged fluorescent images of GFP and MitoTracker are shown. Other conditions are described in Materials and methods.

To determine the mitochondrial targeting signal of HCCR-1, three different sizes of the NH_2_-teminal DNA fragments were subcloned into a pEGFP-N1 expression vector (Fig 3A). Each of the constructs was transfected into COS-7 cells and the mitochondria were stained and examined by confocal microscopy (Fig 3B). HCCR D1-1, which contains NH_2_-terminal segments at positions 1 to 78 residues, was localized diffusely in the cytoplasm and nuclei (Fig 3B, third panel). This distribution is more like that of GFP alone (Fig 3B, top panel). By contrast, the deletion mutants, HCCR D1-2 and D1-3, which contained at position 1 to 110 and 1 to 173 respectively, exhibited a typical mitochondrial pattern, and were overlaid with their mitochondrial staining (Fig 3B, forth and bottom panels); this suggests that the D1-2 region which partially contains LETM1 domain, contains the mitochondrial targeting sequence and D1-1 region alone cannot reach the mitochondria. We also tested these constructs in NIH3T3 and MCF-7 cells, and obtained the same results as in COS-7 cells (Data not shown).

### The D1-2 region of HCCR-1 is required for its mitochondrial targeting

**Figure 4:**
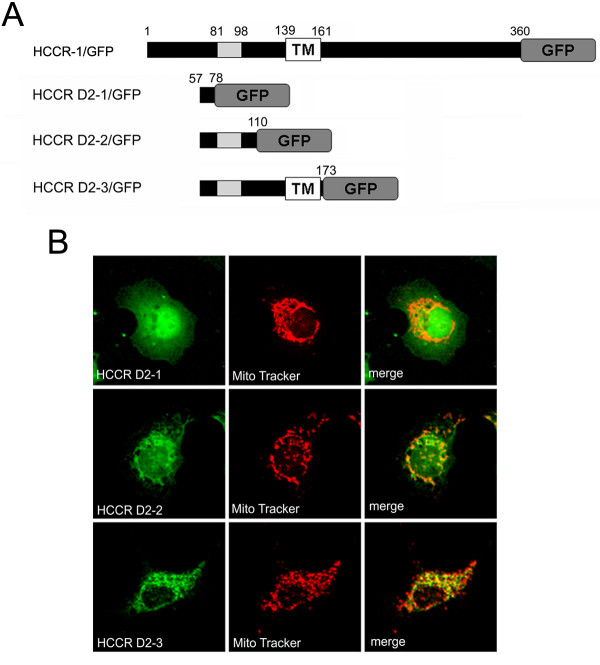
**Mitochondrial targeting of HCCR-1 requires the D1-2 region**. (A) Schematic structure of HCCR-1 deleted mutants which were COOH-terminally linked by the GFP reporter gene. Grey boxes indicate the hydrophobic region. TM boxes indicate the locations of putative transmembrane domain. (B) Expression of deleted mutants in COS-7 cells. COS-7 cells were transfected with the indicated constructs in the expression vectors. Other conditions were same as in Figure 3B.

The 2^nd ^structural analysis of the D1-2 region, using GOR IV secondary structure prediction method [28], identified an α-helical domain from 92 to 110 which belongs to LETM1 domain of HCCR-1 and this domain represented an amphipathic α-helical feature which often plays a role in the mitochondrial targeting signal [25-27]. To further characterize the HCCR-1 targeting signal, the positions 1 to 56 were deleted from HCCR D1-1, -2 and -3 constructs (Fig 4A). Each construct was transfected into COS-7 cells and examined by confocal microscopy (Fig 4B). The HCCR D2-1 construct exhibited a diffuse pattern in the cytoplasm and nuclei resembling that of GFP alone in COS-7 cells (Fig 4B, top panel). The HCCR D2-2 construct contains an amphipathic α-helical domain. This construct also showed a diffuse distribution in cytoplasm and nuclei (Fig 4B, middle panel), which is different from the pattern of HCCR D1-2, and suggests that the NH_2_-terminal region from 1 to 56 is also required for mitochondrial targeting. However, some of the D2-2 mutant was transported to mitochondria (Fig 4B, middle panel), which suggests that this region between 57 and 110 as well as the region from 1 to 56 is involved in the mitochondrial targeting. When the HCCR D2-3 construct was expressed in COS-7 cells, it was not detected in nuclei but in cytoplasm and other organelles (Fig 4B, bottom panel). The D2-3 mutant contains the transmembrane domain which is moderately hydrophobic [18]. This may result in absence in nuclei and reaching into the mitochondria or other organelles [29,30].

Therefore, the mitochondrial localization of HCCR-1 requires its D1-2 region, which partially belongs to the LETM1 domain of HCCR-1. By contrast, D1-1 which contains NH_2_-terminus of HCCR-1 and D2-2 which belongs the LETM1 domain of HCCR-1, were prevented from localizing to mitochondria. This suggests that the D1-1 region may be co-related with the D2-2 region for mitochondrial targeting.

### HCCR D1-2 mutant was exclusively localized at the mitochondria

**Figure 5:**
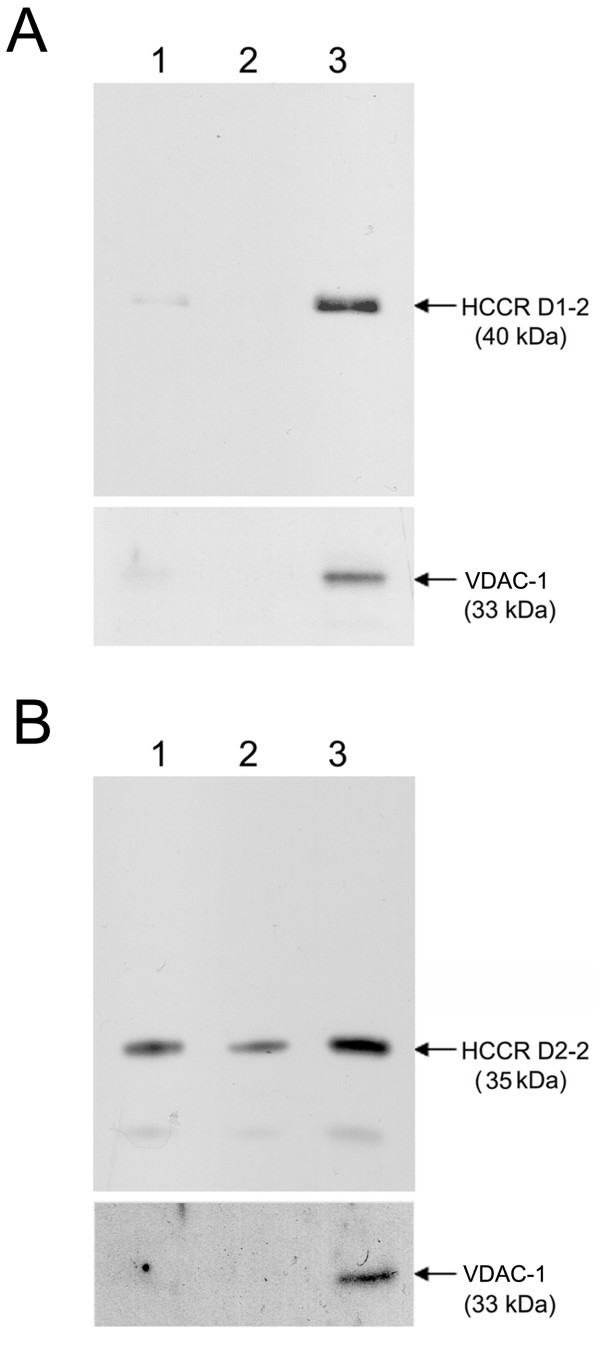
**HCCR D1-2 mutant was exclusively localized at the mitochondria**. COS-7 cells were transfected with HCCR D1-2 (A) and D2-2 (B) which were linked with GFP at its COOH-termini and then incubated for 2 days. The cells were fractionated into the nuclei (lane 1), post-mitochondria (lane 2), and mitochondria (lane3) fractions. Each 10 μg of proteins was separated by SDS-PAGE and analyzed by immunoblotting using antibodies to the GFP epitope (top panel), the mitochondrial protein VDAC-1 (bottom panel).

In order to confirm the previous results obtained by confocal microscopy experiments, COS-7 cells expressing HCCR D1-2 and D2-2 were subjected to subcellular fractionation. Nuclear (Fig 5A and 5B, lane 1), post-mitochondrial supernatant (Fig 5A and 5B, lane2), and mitochondrial (Fig 5A and 5B, lane3) fractions were separated by differential centrifugation, and analyzed by Western blotting using antibodies to the GFP (Fig 5A and 5B, top panel) and the mitochondrial membrane protein VDAC-1 (Fig 5A and 5B, bottom panel). As expected, the deleted mutant D1-2 was exclusively localized to the mitochondrial fraction (Fig 5A), while the D2-2 exhibited all fractions (Fig 5B). These data confirmed that the D1-2 domain is essential for the mitochondrial targeting of HCCR-1.

At the figure 4B and 5B, most of D2-2 and D2-3 mutants are located on mitochondria although some of them are failed to reach to mitochondria. This suggests that HCCR-1 cytosolic form such as HCCR-2 which is deleted at position 1 to 56 amino acids, may exist. This cytosolic form could act as a negative regulator of p53 or could not. However, we propose that this form may not affect the HCCR function because it may also mainly localize at mitochondria.

### Suppression of UVC- or staurosporine-induced apopotosis in HCCR-1 overexpressed cell

**Figure 6:**
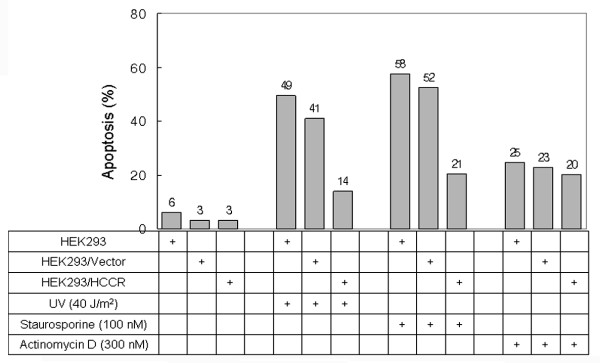
**Suppression of UVC- and stauroeporine-induced apopotosis in HCCR-1 overexpressed cell**. (A) HEK293, HEK293/Vector and HEK293/HCCR-1 cells were exposed to 40 J/m^2 ^of the short-wave length light (UVC) ray, 100 nM of staurosporine and 300 nM of actinomycin D, in respectively. The cells were than stained with propidium iodide and analyzed by FACS. The % of apoptotic cells from each analysis were presented on a bar graph.

To verify the role of HCCR-1 on mitochondrial outer membrane and p53 negative regulation role, we used the HEK293 cell line which stably expresses the vector alone (HEK293/Vector) and V5 tagged HCCR-1 (HEK293/HCCR-1), respectively. The cells were stimulated with the 40 J/m^2 ^of the short-wave length light (UVC) ray, 100 nM staurosporine or 300 nM actinomycin D for trigging apoptosis. After the stimulation, the cells were stained with propidium iodide and analyzed by FACS. The cell number of apoptotic sub G_0_/G_1_-phase in the HEK293/HCCR-1 cells after treatment with UVC or staurosporine was less increased in comparing with that in HEK293 and HEK293/Vector cells, in respectively (Fig 6). The cell number of apoptotic phase in the HEK293/HCCR-1 after treatment with actinomycin D was not significantly changed at all tested cell line (Fig 6). Our flow cytometric data indicate that UVC or staurosporine induced-nuclear apoptosis is retarded in HEK293/HCCR-1 cells, which suggest that HCCR-1 induces HEK293 cells more resistance to UVC-ray and staurosporine-triggered apoptosis, but not in actinomycin D.

We have shown that overexpression of the cDNA encoding HCCR-1 led to the detection of the protein in mitochondira compartment in COS-7, MCF-7 and HEK-293 cells (Fig 2A). However, the previous immunofluorescence study showed that HCCR was predominantly localized in the plasma membrane and cytoplasm of hepatocellular carcinoma [31]. Interestingly, our resent analysis of HCCR mRNA using reverse transcription-PCR and sequencing has identified several HCCR isoforms from various cancerous cell lines and tissues (data not shown). The expression of these isoforms also depended on the cell type and tissues, while some of the isoforms were predicted to be different subcellular organelles. This could be invoked, for example, to account for the generation of the other cellular compartment pool of HCCR.

HCCR-1 has been isolated and identified as a strong oncoprotein with tumorigenic features [13,14]. Interestingly, this protein has been localized to the mitochondrial membrane which has been deeply related to the intrinsic apoptotic signal [1,32]. Here, we showed that the oncoprotein HCCR-1 represses mitochondrial dysfunction caused by its uncontrolled expression. This may prevent the apoptotic signal, block the senescence, and induce cancer development. Therefore, the present data now make it possible to investigate mitochondrial function and mitochondria-mediated apoptosis in HCCR-1 overexpressed cells.

## Conclusion

We report that HCCR-1 is a LETM1 domain homologous protein and is predicted to be a mitochondrial protein. Fluorescence microscopy and fractionation experiments showed that HCCR-1 localizes to the mitochondria in COS-7, MCF-7 and HEK293 cell lines and subcompartamentally at its outer membrane in HEK293 cell lines. Its topology is revealed by the NH_2_-terminus of HCCR-1 which is oriented toward the cytoplasm. We also demonstrated that the HCCR D1-2 domain (amino acids 1–110) is required and sufficient for posttranslational mitochondrial import. Finally, the function of HCCR-1 on mitochondrial membrane suppresses the intrinsic apoptotic signal which is induced by UVC and staurosporine, in respectively.

## Methods

### Cell culture and Transfection

Human embryonic kidney (HEK) 293 (ATCC CRL-1573), COS-7 (ATCC CRL-1651) and MCF-7 breast cancer (ATCC HTB-22) cells were obtained from the American Type Culture Collection (Manassas, VA). HEK293/HCCR-1-V5 cell line [13] which stably expressed V5 tagged HCCR-1 in HEK293 cells, was maintained in DMEM (Gibco, Grand Island, NY) containing 200 μg/ml G418, 10% FBS (Hyclone, Logan, Ut) and 1% PenStrep (Gibco, Grand Island, NY). COS-7 cell line was maintained in RPMI (Gibco, Grand Island, NY) supplemented with 10% FBS and 1% PenStrep. All cells were incubated at 37°C, 5% CO_2_.

### DNA constructs and Transfection

HCCR-1, and its deletion mutants, were amplified using a Peltier Thermal Cycler PTC-200 (MJ Research, MA) with the primers DL1-F (5'-CCGCTCGAGCGGATGGCGCTCTCCAGGGTGTG-3') and FU-R (5'-CGGGATCCCGCGCCTTGTCCCAAGGTAGT-3') for HCCR-1 full open reading frame (ORF), the primers DL1-F and DL1-R (5'-CGGGATCCCCTGACGACCCAAGAAACGAT-3') for D1-1, the primers DL1-F and DL2-R (5'-CGGGATCCCCATATTTGTCTTTATTCTTC-3') for D1-2, the primers DL1-F and DL3-R (5'-CGGGATCCCGGGGGTCCAGAAATGCCTGAT-3') for D1-3, the primers DL2-F (5'-CCGCTCGAGCGGATGTCTTATGTGGTAACCAA-3') and DL1-R for D2-1, the primers DL2-F and DL2-R for D2-2, and the primers DL2-F and DL3-R for D2-3. The primers used in these PCR reactions were synthesized from GenoTech (GenoTech, Daejeon, Korea). Restriction enzyme sites were underlined – single lines for *Xho*I and double lines for *Bam*HI. The PCR cycling parameters were as follows: initial denaturation at 94°C for 2 min; 30 cycles of 30 s at 94°C for denaturation, 30 s at 55°C for primer annealing, and 1 min at 72°C for extension; and final extension at 72°C for 10 min. After amplification, the PCR products were resolved by an agarose gel electrophoresis and purified using Qiagen columns (Qiagen Inc, Valencia, CA). The PCR products were subcloned into the pEGFP-N1 vector (Promega, Madison, WI) at *Xho*I and *Bam*HI sites and sequenced.

For the DNA transfection, all the recombinant plasmids were prepared using Qiagen columns (Qiagen Inc, Valencia, CA) and DNA transfection was performed according to the manufacturer's instructions using LipofectAMINE 2000 reagent (Invitrogen, Carlsbad, CA). Plasmid DNA (0.5 μg) was transfected to COS-7 cells grown on a 6 well dish and the cells were incubated for 24 -28 h before analysis.

### Isolation and subfractionation of Mitochondria

Subcellular fractionation of HEK293/HCCR-1-V5 and COS-7 cells was carried out according to the mitochondria isolation kit (PIERCE, Rockford, IL). Briefly, cells were harvested by centrifuging at 850 g for 2 min and the pellet was suspended with 800 μl of reagent A, and then incubated exactly 2 min on ice. Next 10 μl of Reagent B was added to the suspended solution and incubated for 5 min on ice with vortexing at maximum speed every minute. Then 800 μl of Reagent C was added to the solution and inverted the tube several times to mix. The solution was centrifuged at 700 g for 10 min at 4°C, and the pellet was used for crude nucleic fraction. The supernatant was continuously centrifuged at 12,000 g for 15 min at 4°C and transferred to a new tube for the post-mitochondrial supernatant fraction. The pellet was washed with 500 μl of Regent C, and used for isolation of the mitochondrial fraction.

For the isolation of the mitochondrial membrane fraction, the isolated mitochondria were resuspended in 200 μl of freshly prepared 100 mM sodium carbonate pH 11.5 and incubated on ice for 30 min, and centrifuged at 100,000 g for 30 min at 4°C. The supernatant was used as a mitochondrial matrix fraction. The pellet was resuspended with the same volume (200 μl) of 1X SDS loading buffer and used as a mitochondrial membrane fraction.

### Analysis of Topology

Intact mitochondria were isolated from cultured HEK293/HCCR-1-V5 cells and diluted with MSB buffer (150 mM Potassium acetate, 5 mM Magnesium acetate, 50 mM HEPES pH 7.6, 200 mM Sucrose, 1 mM DTT) and treated with or without 50 μg/ml of proteinase K or proteinase K plus 1% Triton X-100 at room temperature for 30 min. Stop proteolysis was performed by adding 15% (w/v) trichloroacetic acid (TCA, Sigma-Aldrich Corporation, MO) and incubating for 15 min on ice. TCA pellets were collected by microcentrifuging for 5 min at 8,000 g, at room temperature. The supernatants were carefully removed and the pellets were suspended in 30 μl of 1X SDS sample buffer by vortex mixing and subjected to Western blot analysis.

### Western blot analysis

Each fraction was quantified with the BCA Protein Assay Reagent kit (PIERCE, Rockford, IL). Samples in 1X SDS sample buffer were subjected to electrophoresis in a SDS-PAGE and transferred to nitrocellulose by standard procedures. Membranes were blocked with 5% nonfat milk and 0.05% Tween 20 in TBS (Tris-buffered saline, pH8.0) for 1 h at room temperature. After washing with 0.05% Tween 20 in TBS, the membranes were incubated with primary antibodies, anti-V5 (Invitrogen, Carlsbad, CA), anti-GFP (Santa Cruz Biotechnology, Santa Cruz, CA), anti-TOM40 (Santa Cruz Biotechnology, Santa Cruz, CA), anti-TIM23 (Santa Cruz Biotechnology, Santa Cruz, CA), anti-SOD-2 (Santa Cruz Biotechnology, Santa Cruz, CA), or anti-VDAC1 (Santa Cruz Biotechnology, Santa Cruz, CA) antibodies. After washing with 0.05% Tween 20 in TBS, immunoblots were incubated with horseradish-peroxidase conjugated rabbit anti-mouse IgG (Santa Cruz Biotechnology, Santa Cruz, CA) or goat anti-rabbit IgG (KPL, Gaithersburg, MD), and proteins were visualized using enhanced chemiluminescence according to the manufacturer's instructions (PIERCE, Rockford, IL).

### Fluorescence Microscopy

Coverslips were washed with HCl, three times with distilled water and twice with 100% Ethanol. Dried coverslips were then coated with 5 μg/ml Poly-L-Lysine (Sigma-Aldrich Corporation, MO) for overnight at 37°C. COS-7 and HEK293/HCCR-1-V5 cells were seeded onto precoated coverslips in 6 well plates. Then following one day incubation, the cells were transiently transfected using LipofectAMINE 2000 (Invitrogen, Carlsbad, CA). After 24–28 h further incubation, 100 nM MitoTracker (Molecular Probes, Eugene, OR) was added to the medium and incubated for 15 min at the same culture condition and washed three times with 2 ml PBS before fixation. The cells on the coverslips were then fixed with 4% paraformaldyhyde containing 4% sucrose at 4°C for 10 min. To stain V5 tagged HCCR-1, HEK293/HCCR-1-V5 cells were fixed with 100% methanol at -20°C for 1 min and washed three times with PBS and incubated with mouse anti-V5 antibody (Invitrogen, Carlsbad, CA) at a dilution of 1:300 for 1 h at room temperature. They were washed with PBS, Alexa Fluor 488 goat anti-mouse IgG antibody (Molecular Probes, Eugene, OR) at a dilution of 5 μg/ml was applied for 1 h at room temperature. The cells were washed three times with PBS and mounted using ProLong Gold Antifade Reagents (Molecular Probes, Eugene, OR). Fluorescent images were analyzed and taken using a Bio-Rad MRC-1024MP laser scanning confocal microscope (Bio-Rad, Hercules, CA).

### Flow cytometric analysis

The cells were stimulated with the 40 J/m^2 ^of the short-wave length light (UVC) ray and incubated then for 2 days, or with 100 nM staurosporine and 300 nM actinomycin D for overnight, in respectively. After the stimulation, cells were washed two times with ice-cold PBS and stored at -20°C with 5 ml of 70% Ethanol for a day. For the Propidium iodide staining, cells were resuspended and collected by centrifugation at 4°C and washed two times with PBS and then resuspended with 0.5 ml of PBS. 10 μl of RNase (100 μg/ml) were added and the cells were incubated at 37°C for 20 min. After removing RNAs, 1 μl of Propidum iode (50 μg/ml) were added and incubated at 37°C for 10 min for staining. And then analyzed on a BRYTE HS flow cytometer (Bio-rad, Herts, England).

## List of abbreviations used

HCCR, human cervical cancer oncogene; LETM1, leucine zipper-, EF-hand-containing transmembrane protein 1; TOM, translocase of the outer mitochondrial membrane; TIM, translocase of the inner mitochondrial membrane; WHS, Wolf-Hirschhorn syndrome; HEK 293, human embryonic kidney 293; DMEM, Dulbecco's modified Eagle's Medium; PCR, polymerase chain reaction; TCA, trichloroacetic acid; TBS, tris-buffered saline; GFP, green fluorescent protein; PBS, phosphate buffered saline; TM, transmembrane domain; VDAC-1, voltage dependant anion channel 1; SOD-2, superoxide dismutase-2.

## Authors' contributions

GWC, SMS, and SAH performed generation of DNA constructs, transfection and mitochondrial isolation. HKK, SK, and JHY performed fluorescence microscopy and flow cytometric analyses. SYH, TEK and JWK contributed to collecting materials. GWC and JWK participated in the study design and coordination, together with drafting the manuscript. All authors read and approved the final manuscript.

## References

[B1] Hail N (2005). Mitochondria: A novel target for the chemoprevention of cancer. Apoptosis.

[B2] Jezek P, Hlavata L (2005). Mitochondria in homeostasis of reactive oxygen species in cell, tissues, and organism. Int J Biochem Cell Biol.

[B3] Rehling P, Brandner K, Pfanner N (2004). Mitochondrial import and the twin-pore translocase. Nature Reviews Mol Cell Biol.

[B4] Neupert W (1997). Protein import into mitochondria. Annu Rev Biochem.

[B5] Koehler CM, Merchant S, Schatz G (1999). How membrane proteins travel across the mitochondrial intermembrane space. Trends Biochem Sci.

[B6] Geissler A, Chacinska A, Truscott KN, Wiedemann N, Brandner K, Sickmann A, Meyer HE, Meisinger C, Pfanner N, Rehling P (2002). The mitochondrial presequence translocase: an essential role of Tim50 in directing preproteins to the import channel. Cell.

[B7] Yamamoto H, Esaki M, Kanamori T, Tamura Y, Nishikawa S (2002). Tim50 is a subunit of the TIM23 complex that links protein translocation across the outer and inner mitochondrial membranes. Cell.

[B8] Schlickum S, Moghekar A, Simpson JC, Steglich C, O'Brien RJ, Winterpacht A, Endele SU (2004). LETM1, a gene deleted in Wolf-Hirschhorn syndrome, encodes an evolutionarily conserved mitochondrial protein. Genomics.

[B9] Endele S, Fuhry M, Pak SJ, Zabel BU, Winterpacht A (1999). LETM1, a novel gene encoding a putative EF-hand Ca(2+)-binding protein, flanks the Wolf-Hirschhorn syndrome (WHS) critical region and is deleted in most WHS patients. Genomics.

[B10] Johnson VP, Mulder RD, Hosen R (1976). The Wolf-Hirschhorn (4p-) syndrome. Clin Genet.

[B11] Wilson MG, Towner JW, Coffin GS, Ebbin AJ, Siris E, Brager P (1981). Genetic and clinical studies in 13 patients with the Wolf-Hirschhorn syndrome [del(4p)]. Hum Genet.

[B12] Nowikovsky K, Froschauer EM, Zsurka G, Samaj J, Reipert S, Kolisek M, Wiesenberger G, Schweyen RJ (2004). The LETM1/YOL027 gene family encodes a factor of the mitochondrial K+ homeostasis with a potential role in the Wolf-Hirschhorn syndrome. J Biol Chem.

[B13] Ko J, Lee YH, Hwang SY, Lee YS, Shin SM, Hwang JH, Kim J, Kim YW, Jang SW, Ryoo ZY, Kim IK, Namkoong SE, Kim JW (2003). Identification and differential expression of novel human cervical cancer oncogene HCCR-2 in human cancers and its involvement in p53 stabilization. Oncogene.

[B14] Ko J, Shin SM, Oh YM, Lee YS, Ryoo ZY, Lee YH, Na DS, Kim JW (2004). Transgenic mouse model for breast cancer: induction of breast cancer in novel oncogene HCCR-2 transgenic mice. Oncogene.

[B15] Thompson JD, Higgins DG, Gibson TJ (1994). CLUSTAL W: improving the sensitivity of progressive multiple sequence alignment through sequence weighting, position-specific gap penalties and weight matrix choice. Nucleic Acids Res.

[B16] Emanuelsson O, Nielsen H, Brunak S, von Heijne G (2000). Predicting subcellular localization of proteins based on their N-terminal amino acid sequence. J Mol Biol.

[B17] Small I, Peeters N, Legeai F, Lurin C (2004). Predotar: A tool for rapidly screening proteomes for N-terminal targeting sequences. Proteomics.

[B18] Moller S, Croning MD, Apweiler R (2001). Evaluation of methods for the prediction of membrane spanning regions. Bioinformatics.

[B19] Fujiki Y, Hubbard AL, Fowler S, Lazarow PB (1982). Isolation of intracellular membranes by means of sodium carbonate treatment: application to endoplasmic reticulum. J Cell Biol.

[B20] Colombini M (1989). Voltage gating in the mitochondrial channel, VDAC. J Membr Biol.

[B21] Ravindranath SD, Fridovich I (1975). Isolation and characterization of a manganese-containing superoxide dismutase from yeast. J Biol Chem.

[B22] von Heijne G (1994). Signals for protein targeting into and across membranes. Subcell Biochem.

[B23] Diekert K, Kispal G, Guiard B, Lill R (1999). An internal targeting signal directing proteins into the mitochondrial intermembrane space. Proc Natl Acad Sci USA.

[B24] Lee CM, Sedman J, Neupert W, Stuart RA (1999). The DNA helicase, Hmi1p, is transported into mitochondria by a C-terminal cleavable targeting signal. J Biol Chem.

[B25] Gavel Y, Nilsson L, von Heijne G (1988). Mitochondrial targeting sequences. Why 'non-amphiphilic' peptides may still be amphiphilic. FEBS Lett.

[B26] Roise D (1997). Recognition and binding of mitochondrial presequences during the import of proteins into mitochondria. J Bioenerg Biomembr.

[B27] Waltner M, Hammen PK, Weiner H (1996). Influence of the mature portion of a precursor protein on the mitochondrial signal sequence. J Biol Chem.

[B28] Garnier J, Gibrat JF, Robson B (1996). GOR method for predicting protein secondary structure from amino acid sequence. Methods Enzymol.

[B29] Kanaji S, Iwahashi J, Kida Y, Sakaguchi M, Mihara K (2000). Characterization of the signal that directs Tom20 to the mitochondrial outer membrane. J Cell Biol.

[B30] Horie C, Suzuki H, Sakaguchi M, Mihara K (2002). Characterization of signal that directs C-tail-anchored proteins to mammalian mitochondrial outer membrane. Mol Biol Cell.

[B31] Yoon SK, Lim NK, Ha SA, Park YG, Choi JY, Chung KW, Sun HS, Choi MJ, Chung J, Wands JR, Kim JW (2003). The human cervical cancer oncogene protein is a biomarker for human hepatocellular carcinoma. Cancer Res.

[B32] Coultas L, Strasser A (2003). The role of the Bcl-2 protein family in cancer. Semin Cancer Biol.

